# Ethical and management dilemmas in the care of the newborn at the limit of viability: a narrative review

**DOI:** 10.25122/jml-2026-0020

**Published:** 2026-02

**Authors:** Claudiu Voic, Laura Georgiana Caravia, Alina Maria Borcan, Ioana Maria Toplicean, Madalina Timircan, Zoran Laurentiu Popa, Cezara Maria Mureşan, Melinda Ildiko Mitranovici, Elene Bernad

**Affiliations:** 1 Doctoral School, Victor Babes University of Medicine and Pharmacy, Timisoara, Romania; 2 Division of Cellular and Molecular Biology and Histology, Department of Morphological Sciences, Carol Davila University of Medicine and Pharmacy, Bucharest, Romania; 3 Department of Microbiology, Prof. Dr. Matei Balș National Institute for Infectious Diseases, Bucharest, Romania; 4 Carol Davila University of Medicine and Pharmacy, Bucharest, Romania; 5 Department of Biology-Chemistry, Faculty of Chemistry, Biology, Geography, West University of Timisoara, Timisoara, Romania; 6 Department of Obstetrics and Gynecology, Faculty of Medicine, Victor Babes University of Medicine and Pharmacy, Timisoara, Romania; 7 Center for Laparoscopy, Laparoscopic Surgery and In Vitro Fertilization, Faculty of Medicine, Victor Babes University of Medicine and Pharmacy, Timisoara, Romania; 8 Clinic of Obstetrics and Gynecology, Laparoscopy, In Vitro Fertilization and Embryotransfer Research Center, Pius Brinzeu County Clinical Emergency Hospital, Timisoara, Romania; 9 Doctoral School of Medicine and Pharmacy, George Emil Palade University of Medicine, Pharmacy, Science, and Technology of Targu Mures, Targu Mures, Romania; 10 Department of Obstetrics and Gynecology, Emergency County Hospital Hunedoara, Hunedoara, Romania; 11 Center for Neuropsychology and Behavioral Medicine, Victor Babes University of Medicine and Pharmacy, Timisoara, Romania

**Keywords:** preterm infants, limit of viability, ethical dilemmas, healthcare professionals, decision-making, NICU, Neonatal Intensive Care, IUGR, intrauterine growth restriction, TOP, Termination of pregnancy, ICR, intact cord resuscitation, ISUOG, International Society of Ultrasound in Obstetrics and Gynecology, CPAP, continuous positive air pressure

## Abstract

There are variations among healthcare providers regarding the gestational age considered the threshold of viability. Currently, the World Health Organization sets the lower limit of viability at 22 weeks of gestation, or a birth weight of 500 g, or a birth length of 25 cm. Neonates are not autonomous. The present study aimed to evaluate the factors involved in healthcare professionals’ management decisions in cases of neonates born at the margins of viability. A narrative review was conducted, including articles published between 2005 and 2025. We included 55 manuscripts and used the SANRA quality scale for assessment. The various approaches currently used worldwide raise concerns about their potential impact on the quality of care provided to these infants. We explored survey-based evidence regarding perceptions of the limit of viability and therapeutic decisions that raise ethical issues. The lack of coherent national guidelines and legislation represents an important burden for our healthcare system. Clinicians are faced with a dilemma regarding the correct management of infants at the limit of viability: whether to prolong treatment despite possible neurological impairment or to indicate discontinuation of treatment. Prenatal counseling for parents before delivery, along with a multidisciplinary approach, is required.

## Introduction

The limit of viability represents a major challenge in the management of newborns worldwide. There are variations among health care providers regarding the gestational age considered the threshold of viability. Survey questions on perceptions of the limit of viability have been developed to identify respondents’ views about the optimal gestational age threshold for viability [1]. The management and treatment of these extremely preterm newborns remain controversial and raise important ethical concerns [2].

Another dilemma arises when using the term “viability” in clinical practice, because there is a distinction between “pregnancy viability” and “fetal viability”. This issue surrounding the term “viable” has contributed to confusion regarding pregnancy and leads to controversy, particularly when courts and legislatures appropriate medical terminology. Clear definitions, free from controversy, are required [3].

In current practice, obstetricians use the terms viability or viable in two contexts:

1. At the time a fetus can survive outside of the uterus, meaning fetal viability. It depends on fetal organ maturity, linked to gestational age, and on the ability to live outside the uterus.
2. When an embryo has a detectable heartbeat, which is pregnancy viability in contrast with a failed pregnancy [3].

The viability of a neonate is influenced by several factors, in addition to fetal weight, such as the absence of severe structural anomalies, infection during pregnancy, and oligoamnios [3].

The definition of infants born at the limits of viability differs between countries, depending on the medical conditions in which the infant is born, the development of neonatal intensive care, and its organization [4]. Also, the definition of the limits of viability is not quite clear. There are several aspects to consider: the gestational age and/or birth weight at which the human fetus has the capability of survival outside the uterus, as well as the gestational age and/or birth weight at which infants survive to discharge home from the hospital. There are significant differences between countries, also based on each country’s resources to care for infants born at the limits of viability. Currently, the World Health Organization (WHO) sets the lower limit of viability at 22 weeks of gestation, or 500 g birth weight, or 25 cm of birth length. In our country, the limit is set at 24 weeks without including perinatal counseling with the participation of a neonatologist in the management of the pregnant woman, also excluding ethical issues [4].

In Taiwan, a survey of periviable infants born at 22–25 weeks of gestational age reported varying survival rates across hospitals, ranging from 49.7% to 86.2% [5]. In Israel, several factors influence neonatologists’ decisions regarding the resuscitation of preterm infants born at 22–24 weeks of gestation, with religious beliefs being one of them [6,7].

Children are not autonomous and lack the capacity to make decisions about their medical care. Therefore, parents and the medical team make decisions on behalf of the neonate. In addition, other factors are discussed, for example, legislative bodies, ethics committees, religious leaders, and political interest groups regarding resuscitation and life-sustaining therapies [8]. Neonatal intensive care (NICU) for infants born at 22–24 weeks has developed in the past three decades, but outcomes remain highly variable between countries, even centers. The increasing costs of care for survivors have also been taken into consideration [9]. Advances in neonatal care have gradually lowered the limit of viability over the last few decades. However, extremely premature neonates may survive with long-term neurodevelopmental disabilities. Ethics has an extremely important role in periviable birth management, because it has been observed that at 22 weeks, survival varies from 1 to 15% and is associated with profound disabilities in survivors [10]. At the same time, the autonomy of parents’ wishes should be respected, legal aspects must be taken into account, and informed consent should be obtained before any intervention [10]. The issue of the borderline of viability has also generated ethical controversies, particularly when individual centers implement their own policies [11].

Statistically, in our country in 2024, the mortality of newborns at the limit of viability increased significantly. From a legal perspective, there is a grey zone between 22 weeks of gestation or 500 g of birth weight and 27 weeks and 6 days in Romania. If the newborn is born alive, the event is legally considered a birth; if the newborn is dead, it is considered a miscarriage. This situation highlights the complexity of managing such cases, starting with antenatal counseling.

The present manuscript aimed to evaluate the factors involved in healthcare professionals’ management decisions in cases of neonates born at the margins of viability. Important issues that are not included in many current guidelines were evaluated in our manuscript, including ethical issues, cultural differences across countries, and legislation. The importance of healthcare professionals’ training is also highlighted in the management of these neonates, together with the need for a multidisciplinary strategy. Various approaches currently used across the world raise concerns about their potential impact on the quality of care provided to these infants and add emotional stress to those involved.

## Material and Methods

Relevant studies published in PubMed, Google Scholar, and Cochrane were selected as the primary databases for the search. The authors searched for relevant manuscripts using the following specific keywords: “preterm infants,” “limit of viability,” “survey questionnaire,” “healthcare professionals,” and “decision-making,” covering articles published over the last few decades. Using Boolean operators, the search strategy presented in Table 1 was developed.

**Table 1:** Boolean search strategy used for literature retrieval

AND	OR	NOT
Healthcare professionals AND decision-making	Preterm infants OR limit of viability	Limit of viability in neonates NOT children

The inclusion criteria were full-text articles written in English, appropriate study designs aligned with the purpose of our study, addressing preterm deliveries at the limit of viability, informative articles regarding ethical issues, or clinical decision-making for neonates at the limits of viability, based on short- and long-term outcomes. Initially, 767 titles were generated. The exclusion criteria were: duplicates; studies in a language other than English; and non-peer-reviewed material, such as books and editorials. Also, studies that were inaccurate or inappropriate, based on title and abstract screening, or manuscripts with an inappropriate study design after full-text screening were excluded. After rigorous evaluation using the Scale for the Assessment of Narrative Review Articles (SANRA) quality assessment tool [12], 55 articles were included in the review. We opted for the format of a narrative review because the research question addressed in our manuscript, healthcare professionals’ management decisions regarding neonates born at the limit of viability, is too broad for a systematic review. In addition, a narrative review allows the identification of knowledge gaps and provides an educational overview of the topic.

## Discussions

We identified discrepancies among physicians based on variations in reported outcomes and prognostic assessments. Some physicians were more optimistic, others more pessimistic, while in some studies their opinions were not clearly measured. In addition, several factors were found to influence prognosis. We included articles that evaluated the opinions of specialists, including obstetricians, neonatologists, and midwives. Other manuscripts assessed the perspective of only one type of specialist, for example, only obstetricians or only neonatologists.

Different factors influence treatment decisions [8]:

- demographic data such as age, gender, specialty (obstetrician, neonatologist, midwife) of the responders, experience, and training
- diagnosis, clinical context, maternal/patient factors: age, comorbidities, fetal severe anomalies
- ethical issues: parents’ personal beliefs, moral and religious context, physician-patient relationship
- environmental-related factors: country or hospital size, resources, patient location, rural or urban.

### a. Discrepancies based on demographic characteristics of the study participants

Comparative demographic characteristics of respondents were considered in the study by Nuthalapaty *et al.*, which included 580 respondents. [1]. The perceived threshold for the limit of viability was not significantly influenced by practitioner age, years of experience, or place of work. However, significant differences were reported between men and women, with a trend toward a lower gestational age threshold among males (*P* = 0.005). The limits of viability varied, but the majority of practitioners identified 23–24 weeks of gestation [1]. Also, it has been observed that ongoing training and education of midwives and obstetric nurses are essential to ensure the best outcomes [13].

In a survey on 155 responders regarding attitude toward extremely preterm birth, Bucher *et al*. reported important variations [14]. Differences between obstetric and neonatal health care professionals were found in the prioritization of areas, including medical technology used, parental involvement in decision-making, and infant outcomes. For example, nurses mentioned the lack of services for people with disabilities more often than physicians did. In addition, nurses and neonatologists considered that end-of-life decisions should not be made by parents [14]. Therefore, we agree with Gallagher that multidisciplinary training may be useful in developing a personalized approach and may reduce potential misalignment within and between professional groups [15].

### b. Diagnosis clinical context

Clinical context, as well as maternal and fetal morbidities leading to variations in outcomes, are important factors in healthcare professionals’ decision-making. Even so, there are variations between physicians regarding management. In his research, Di Stefano *et al*. investigated the lower and upper thresholds of gestational age at which healthcare professionals would offer treatment using an online survey. Most participants understood the limits of viability for an infant who can survive without accounting for comorbidities. Active treatment has been offered for infants <23 weeks’ gestation [2]. We consider that perinatal counseling and decision-making should focus on the newborn, rather than solely on fetal and maternal antenatal management. However, life support in the NICU should focus on the quality of life in the surviving infant, whose outcome can rarely be predicted accurately [8].

In the study by Chen *et al.*, 168 periviable infants were included, while those with congenital anomalies were excluded. They observed a younger gestational age among participants, and some infants presented intrauterine growth restriction (IUGR). Other risk factors encountered were cesarean delivery and low blood pH at birth. Treatment with surfactant was associated with better outcomes of peri-vital infants (*P* < 0.05 for each). Long-term follow-up is required [5]. With the help of various medical therapies, such as antenatal steroids, resuscitation, and continuous positive airway pressure (CPAP), chronic lung disease and other disabilities may be prevented [8]. These observations help improve the quality of life of children born at the limit of viability and support the importance of multidisciplinary perinatal counseling.

The common morbidities observed by Shucl *et al*. in a cohort of 187 eligible infants born at 22–23 weeks were necrotizing enterocolitis, patent ductus arteriosus, sepsis, and severe intraventricular hemorrhage [16]. In the review by Al Hazani *et al*. on preterm infants born at 23–25 weeks of gestation, at the limit of viability, which included 97 infants, the major neonatal morbidities were severe brain injury, severe retinopathy, and bronchopulmonary dysplasia [17]. In this regard, we can conclude that personalization is necessary in prenatal parental counseling [18]. However, NICU healthcare professionals have several criteria when discussing the withdrawal of life-sustaining therapies [19]. The resources and level of development of neonatology services will also influence the long-term outcomes of children born at the border of viability [20]. In case of resuscitation, if the heart rate is still undetectable, it is reasonable to discuss the possibility of terminating this procedure and inform the family after approximately 20 minutes. Other perinatal circumstances, such as pre-existing illness, or gestational age, will shape the decision [21].

Antenatal treatment and information shape the course of pregnancy in cases of threatened prematurity on the borderline of viability. The therapeutic measures were discussed with the parents, and 57 % of clinicians said they would advise parents to seek intensive care treatment for the child, with the option to end treatment in the event of serious complications. 84 % of them offered joint counseling with neonatologists [22].

Genomic sequencing provides a powerful tool to identify health conditions before they develop in patients who manifest symptoms of monogenic disease. This could have widespread availability in pediatric care. However, many technical, clinical, ethical, and societal challenges should be addressed before such technology is widely deployed in pediatric practice, including in newborns, for both diagnosis and screening [23].

In a survey of neonatologists in the United States, it was reported that the majority preferred “to see what the infant looked like” in the delivery room before providing full resuscitation. The “gray zone” for delivery room resuscitation seems to be between 500 and 600 g birth weight and 23 and 24 weeks of gestation. The attitude of neonatologists toward infants born in that “gray zone,” based on perceptions in the delivery room, may be misplaced [24]. Compared with previous studies, Gallagher found a lower threshold reported for resuscitation in the UK, in favor of active treatment for infants <23 weeks’ gestation [25].

In a retrospective study on 78 infants born at 22-24 weeks, the survival rates were 40.0% at 22 weeks and 50.0% at 24 weeks. The major cause of death was failure of response to surfactant. Other observations were a low Apgar score, intraventricular hemorrhage (≥III), and sepsis that led to death. Even among those who survived, neurological outcomes remained poor [26].

Long-term neurodevelopmental disabilities should also be taken into consideration, even when survival occurs (ranging from 1–15% at 22 weeks and 8–54% at 23 weeks). Also, the wide range of survival indicates that cooperation between obstetricians and neonatologists is optimal for counselling of parents before delivery [10].

There are complex legal questions regarding extremely premature neonates with poor prognosis that could generate distress in the neonatal intensive care unit. A high proportion of survivors develop disabilities. In some countries, at the parents’ request, neonates at the limit of viability receive palliative care instead of active management. Some level of consensus may be achieved, though disagreements persist over potential outcomes, cultural perspectives, and available resources. The most common controversies concern the threshold for viability. Another important debate concerns the limit of acceptability for healthcare professionals to withhold life-prolonging therapies at parental request. While in countries such as Australia and Canada, proactive management is generally provided for infants born after 24 weeks of gestation, in other countries, the threshold is higher. For example, in the Netherlands, the upper threshold is 26 weeks, while in China, it has been reported to be 28 weeks. There are ongoing arguments regarding the resuscitation of extremely premature infants. However, these debates should primarily focus on what is in the child’s best interests [27,28].

Termination of pregnancy (TOP) may be considered in cases of congenital malformations, even when the fetus may already be viable. In developed regions such as Flanders, Belgium, healthcare professionals have shown a relatively high degree of tolerance toward late TOP, according to a survey including 117 respondents. Legal aspects in each country must be taken into account [29]. Based on these observations, different clinical contexts lead to different attitudes of doctors towards patients at the limit of viability. Sometimes in the same context, the attitude is different. All these aspects need to be aligned and included in future clinical guidelines.

### c. Ethical issues

In Israel, a study including 127 active neonatologists reported that most believed resuscitation and full treatment at birth are against the best interests of infants born at 22–24 weeks of gestation. Personal values and concerns about legal issues were considered to influence decision-making, along with respondents’ gender and parents’ wishes [7].

Appropriate delivery room care and the resources used for resuscitation have also been evaluated for infants born at 22–26 weeks of gestation. In the study by Arbour *et al*., 672 neonatologists in the United States were invited to participate, and 180 responded [30]. The factors with the greatest impact on decision-making at 22 weeks of gestation included outcomes based on population data (79%), parental wishes (65%), and quality-of-life considerations (63%). The therapies most frequently applied were resuscitation, surfactant administration in the delivery room, and establishment of vascular access [30].

The so-called “gray zone” is characterized by prognostic uncertainty. The fetus and newborn cannot exercise their rights, so the parents and clinicians make the decision. We agree that perinatal interventions must be discussed with the parents, whose choice, after detailed information, must be respected, as recommended in the International Federation of Gynecology and Obstetrics (FIGO) guidelines described by Vidaeff [31]. In practice, however, guidelines often do not provide clear recommendations and remain somewhat ambiguous; in such cases, ethics committees may guide legal and ethical aspects [31].

Preterm infants depend on parents and clinicians to make therapeutic decisions that may affect them for a lifetime, ideally in their best interest. Not only survival, but also neurodevelopmental and other relevant outcome measures need to be discussed during prenatal counselling. Recommendations and guidelines must take into consideration these aspects, which are currently arbitrary [20,32]. According to the American Academy of Pediatrics, the Born-Alive Infants Protection Act requires reporting all cases in which infants were allowed to die [33].

A survey of 138 clinicians revealed considerable variation in resuscitation practices amongst different neonatal care providers [34]. Also, potential disagreements between parents and professionals regarding the attitude for resuscitation at the limit of viability are common. Written informed consent about mortality and morbidity can lead to overestimation of risk, even among doctors, while in parents, these informed consents subtly shape the decisions that follow [35].

End-of-life decisions for neonates with an adverse prognosis raise ethical and legal issues. Of the 260 eligible physicians, 62 responded to an anonymous survey and chose to continue treatment in neonates with adverse outcomes. The most important predictors of physicians’ attitudes were religiousness and belief in the Greek legal system reform [36]. There is strong evidence for the development of interventions for end-of-life decision-making, which should be carefully addressed [37].

In a cross-sectional study including 2,116 participants, only 17% reported being aware of the recommendations regarding euthanasia and withdrawal of life-prolonging treatment for neonates, indicating a low level of knowledge [38]. Neonatal end-of-life care should focus on supporting terminally ill newborns and their families while minimizing suffering at the end of the neonate’s life [39]. It is very important to acknowledge these aspects to avoid potential conflicts both among clinicians and between clinicians and parents during the decision-making process regarding the withdrawal of life-support treatment in preterm infants born at the limits of viability [14]. In some countries, preterm infants are systematically undervalued compared with older patients, and healthcare professionals may accept a family’s refusal of resuscitation even when they believe that resuscitation would be in the patient’s best interest [40]. According to the study by Lavin *et al*., only minor differences were found between healthcare providers, and there was a relatively consistent approach among professional groups regarding the recommendations that should be made to parents about the resuscitation of newborns at the limit of viability [41].

Disagreements between parents and healthcare professionals regarding attitudes toward resuscitation at the limit of viability were also observed in the study by Apostolidi *et al*. [42].

There is ongoing debate about how to improve life-sustaining treatment, and promising early interventions involving communicative musicality have shown improvements in preterm infant development in the NICU [43].

The acceptability and bioethical justification of active medical treatment in extremely preterm or critically ill neonates among healthcare professionals in Greek hospitals have contributed to improving the quality of neonatal intensive care [44]. Perinatal care demands a delicate balance between parents’ wishes, biological feasibility, and clinicians’ responsibilities and the prospects of an acceptable long-term outcome. Informed consent based on consensus will facilitate greater communication between parents and clinicians [45]. Also, in the Netherlands, the guideline lacks recommendations on content regarding decision-making counselling. Dutch perinatal professionals would prefer a protocolized counselling, including outcome statistics [46].

A significant majority of parents believe that attempts should be made to save all infants, regardless of the infant’s condition or birth weight, compared with only 6% of healthcare professionals who support this view. In contrast to parents, clinicians have considered the economic costs to society, which should be a factor in making this decision. However, most respondents agree that parents and physicians should make the final decision together, highlighting the need for shared decision-making that combines the physician’s medical expertise with parents’ wishes [2,14].

Prenatal counseling at the limits of viability should follow guidelines and recommendations based on evidence-based information. A standardized approach is preferred, but it should not be too rigid; physicians should personalize counseling and provide the specific information that parents need [2].

Different environmental factors, such as cultural, religious, and socioeconomic factors, can influence ethical principles across countries. The decision-making process should integrate parental choice, while healthcare professionals can provide recommendations regarding neonatal resuscitation whenever possible before birth. Currently, guidelines regarding periviable neonates remain vague. The obstetric and neonatal teams should therefore offer a coherent and balanced plan of care, adjusted to local standards and the availability of neonatal support [31].

### d. Environmental-related factors

Within the past decade, there has been a shift in the “gray zone” from 23–24 to 22–23 weeks of gestation. Resuscitation practices for these infants are not standardized. Variability in available resources may contribute to differences in outcomes for periviable infants [30]. There are also notable differences between level III maternity centers and level II or I units [47].

Two main trends can be observed: countries that advocate active treatment for infants born at 22+0 weeks of gestation, such as the United States and the United Kingdom, and countries that more frequently offer palliative care, such as France. However, in almost all countries, the family’s wishes are taken into account [48]. In a study including 163 very premature and extremely preterm newborns hospitalized in neonatal intensive care units, outcomes were evaluated, and the authors observed the use of off-label medication in 60.5% of cases and off-license drugs in 25.3% of newborns. Most deaths occurred due to septic shock, while the main morbidities were related to respiratory distress [48].

In low- and middle-income countries, it is necessary to identify key gaps in maternal and child health that are influenced by socioeconomic, cultural, and healthcare system factors. Practical methodologies are essential for improving child health outcomes [49].

Although the WHO recommends family-integrated newborn care for hospitalized preterm and low-birth-weight infants, in some countries, such as Ethiopia, this approach was found to be difficult to implement. While it was considered conceptually acceptable (74%) and ethically appropriate (88%), its implementation was constrained mainly by limited organizational infrastructure and insufficient NICU space [50].

Charafeddine, in a survey including 328 physicians from developing countries, reported that most respondents considered infants with a gestational age of less than 25 weeks (78%) and a birth weight of less than 800 g (89%) to be nonviable [51].

In a review of clinical guidelines, the authors concluded that differences in the types of data included represent one potential source of variation in viability thresholds within this “ethical gray zone.” Ethical frameworks, clinical considerations, counseling practices, therapeutic management, outcomes, and available data form the basis of these inconsistencies. Ten guidelines lacked any ethical component, even though ethical considerations may significantly influence resuscitation thresholds. These aspects should therefore be clarified [52]. Such issues may also contribute to moral distress among neonatologists [53].

These findings are summarized in Table 2.

**Table 2:** Variability in the limits of the viability threshold

Reference	Country	Ethical issues	Active treatment	Withdrawal of treatment	Comments
**Nuthalapaty *et al***. [1]	50 states and 13 countries			At lower threshold in male responders	Limits of viability 23-24 weeks
**Di Stefano** [2]	UK	protocolized counselling	Active treatment for infants <23 weeks’ gestation		lacks recommendations <23 weeks.
**Chen** [5]	Taiwan		Active treatment with surfactant		<23 weeks
**Sperling** [7]	Israel	Personal values and concerns about legal issues affect decision		Best interest	22-24 weeks
**Ferrand** [8]	Of 4,723 articles, 73 were included in this review (multiple countries)		Steroids, resuscitation, CPAP		Perinatal counseling must focus on the newborn, not only on antenatal management
**Bucher** [14]	Switzerland		Medical technology used, parental involvement in decision-making, and infant outcomes.	Withdrawal of life-support treatment	Differences between obstetric and neonatal health care professionals: Minimize suffering.
**Gallagher** [15]	UK		Active treatment		Before 23 weeks
**Schuler** [20]	Review in the United States	Relevant outcomes should be considered.			Arbitrary recommendations
**Schneider** [22]	Germany		Intensive care treatment		Threshold variability
**Singh** [24]	USA			“to see what the infant looked like.”	23-24 weeks
**Arbour** [30]	United States		Resuscitation, surfactant administration, and vascular access.	Outcomes, parental wishes, and quality of life measures	22 weeks
**Vidaeff** [31]	FIGO recommendations	Legal issues			Guidelines are vague
**Aso** [34]	Nigeria			Variation in resuscitation	Variability in threshold
**Papadimitriou** [35]	Italy			Disagreements between clinicians and parents	Variability in threshold
**Chatziionnidis** [36]	Greece	Ethical and legal issues			Religion-based influence
**Beltran** [39]	USA			Withdrawal of life-support treatment	Minimize suffering
**Janvier** [40]	Canada	Ethical and legal issues		Withdrawal of life-support treatment	Family’s refusal of resuscitation
**Apostolidi** [42]	Switzerland	Ethical and legal issues		Resuscitation withdrawal	Disagreements between parents and professionals
**Haslbeck** [43]	Switzerland	Ethical and legal issues		Withdrawal of life-support treatment	Who should make the final decision
**Dagla** [44]	Greece		Active medical treatment		Extremely preterm or ill neonates
**Ferreira** [48]	Brazil		Active treatment versus palliative care		Depends on the country
**Charateddine** [51]	Lebanon			Non-viable	≤25 weeks
**Chen** [52]	United States	Inconsistencies in ethics, clinical reasons, and others			Variability in threshold

Abbreviations: UK, United Kingdom; USA, United States of America; CPAP, continuous positive air pressure.

### Future perspective

Efforts have been made to identify new therapies that could lower the limit of viability of newborns while improving their quality of life. Recent breakthroughs in the development of extracorporeal neonatal life support using artificial womb technology have renewed ethical discussions on this topic [6].

Regarding cord clamping timing, the study by Hocq *et al*. evaluated the potential use of intact cord resuscitation (ICR) in periviable preterm newborns. This approach can be performed using a mobile resuscitation trolley. Among the 18 infants who underwent this innovative intervention, the time to cord clamping increased significantly without complications. No infant deaths or cases of hypoxic respiratory failure were directly attributable to ICR. Hemoglobin level was higher in the ICR cohort than in the pre-implementation group. These findings suggest that ICR is a feasible option for very preterm children. Special attention should be paid to the temperature management. As an inconvenience, multidisciplinary training is required [54].

In decisions regarding life-saving treatments in newborns, pain assessment must also be considered. There are disciplinary differences in beliefs regarding the recognition of pain in infants. Many clinicians consider limiting suffering to be more important than eliminating pain. They emphasize that alleviating suffering and avoiding chronic distress for neonates and their families may be more pressing concerns than short-term physical pain [55].

Another effective intervention that may improve the health and development of preterm infants is kangaroo care. However, parents’ knowledge and experience with this procedure remains low. The successful implementation of kangaroo care relies on adequate training of both healthcare professionals and parents [56].

Monitoring technologies are also evolving. Wired vital-sign monitoring systems and wireless technologies may represent future alternatives for monitoring infants at the limits of viability in the NICU. Current monitoring systems are not always satisfactory for clinicians or parents. In a survey of 1,141 respondents, 52% reported problems such as interference with skin-to-skin care, tangled wires, and skin irritation [57]. In addition, mobile health applications have the potential to transform healthcare by making it more accessible, cost-effective, and efficient [58]. A new therapeutic avenue was proposed by Haslbeck *et al*., who demonstrated that music interventions can reduce heart rates in very preterm infants and may improve neurodevelopmental outcomes [59].

### Strengths and limitations

The strongest aspect of our review is its worldwide perspective. The limitation of the study is the potential discrepancy between methodologies used across different research, different study designs, or variations in the outcomes tracked. Interpersonal communication and a multidisciplinary approach are not easily highlighted. These results may not be generalized to the population. Our study is, to our knowledge, the first on prenatal counselling in Romania. The manuscript also highlights differences in current counseling practices, including variations between obstetricians and neonatologists.

The limitation of the study lies in the breadth of the manuscript approach. It was not possible to provide a unified strategy that accounted for diagnosis, outcomes, ethical considerations, and the personal practices of healthcare professionals. Furthermore, the choice of a narrative review format may limit the methodological strength of the study. Nevertheless, professional guidelines should be developed that incorporate and promote ethical principles.

The neonatal mortality rate in Romania has reached 6.6 per 1000 live births, the highest in Europe. This situation is partly due to the lack of medical equipment in hospitals, the shortage of qualified personnel, and insufficient training opportunities for healthcare professionals. There are also major disparities between level I and level III hospitals, as well as between different regions of the country.

From a clinical perspective, the novelty of our review lies in highlighting opportunities for improvement in the perinatal management of infants born at the limit of viability and in prenatal counseling. Also, training the professionals involved and providing them with medical equipment would lead to a standardization of the therapeutic approach. This fact, correlated with improved legislation and the inclusion of ethical regulations in current clinical guidelines, would improve the survival of newborns at the limit of viability.

The risks of short-term and long-term neonatal complications following periviable births should be presented accurately and objectively. Further studies regarding preferred prenatal counselling and its implementation are necessary.

Variation in prenatal counseling may be in the patient’s best interest when it reflects individual maternal or fetal characteristics or parental beliefs. However, when such variation results from unclear background information, insufficient organizational support, or inappropriate personal practices of healthcare providers, it does not serve the patient’s best interest. The development and implementation of a nationally supported framework may improve the quality of prenatal consultations while still allowing appropriate individualization of care.

## Conclusion

Among traumatic experiences, losing a child is one of the most painful and may have lifelong repercussions. The lack of coherent national guidelines and legislation represents a significant burden on our healthcare system. At the same time, newborns at the limit of viability have a high risk of neurological impairment, which may lead to the difficult decision of discontinuing treatment. Our review highlighted variation among healthcare professionals in the management of newborns at the limit of viability and potential factors underlying these variations. Based on our findings, a new avenue for perinatal counseling may emerge.

Prenatal counseling for parents before the delivery of a premature infant provides the opportunity to establish a trusting relationship and an appropriate environment for decision-making regarding neonatal resuscitation. More protocolized counselling, with supportive material, is preferred. The uncertainty that surrounds many decisions in the treatment and resuscitation of infants born at the limits of viability creates a situation in which joint responsibility for decision-making between parents and physicians is vital.

Currently, when an infant’s gestational age is considered nonviable, the neonatologist is not always involved in perinatal counseling. The presence of a neonatologist could be helpful, as they can better explain the rationale behind non-active management and provide comfort care for live-born extremely immature infants. This further supports the importance of a multidisciplinary approach.
